# DEVELOPING A PAIN IDENTIFICATION AND COMMUNICATION TOOLKIT FOR FAMILY CAREGIVERS OF PERSONS WITH DEMENTIA

**DOI:** 10.1093/geroni/igz038.2636

**Published:** 2019-11-08

**Authors:** Catherine Riffin, Karl Pillemer, Keela Herr, Emily Petti, Cary Reid

**Affiliations:** 1 Weill Cornell Medicine, New York, New York, United States; 2 Cornell University, Ithaca, New York, United States; 3 University of Iowa, Iowa City, United States

## Abstract

Best practice guidelines have emphasized the importance of routine pain assessment of older persons with dementia (PWD), yet pain remains severely underdetected and undermanaged in this population. Training family caregivers in observational pain assessment and subsequent communication of the assessment results to a healthcare provider has the potential to help improve pain management among PWD. The goal of this presentation is to describe the approach to developing, refining, and pilot testing the Pain Identification and Communication Toolkit (PICT), a novel intervention to help family caregivers recognize pain symptoms in PWD and communicate those symptoms to healthcare providers. Guided by self-efficacy theory and empirical research on dementia and pain communication, this presentation will detail the approach to developing the PICT intervention manual and delineate its major components, including modules that prepare caregivers to: a) recognize and differentiate pain from dementia symptoms, b) administer an observational pain assessment tool, c) communicate effectively about pain symptoms, and d) plan steps to take when pain is detected. The presentation will report results from the process by which preliminary versions of the PICT manual were refined, including iterative field-testing with a sample of racially and ethnically diverse caregivers of community-dwelling PWD and healthcare providers. Results suggest that the development of PICT represents a useful step in addressing the underdetection and undermanagement of pain in PWD, and can pave the way for future intervention research on caregivers’ initiation of pain-related communication with healthcare providers.

